# Mesh abdominoplasty for rectus diastasis in women and men

**DOI:** 10.1007/s10029-021-02461-1

**Published:** 2021-08-03

**Authors:** G. A. Dumanian, S. Moradian

**Affiliations:** Division of Plastic Surgery, Department of Surgery, Northwestern Feinberg School of Medicine, 675 N St. Clair, Suite 19-250, Chicago, IL 60611 USA

**Keywords:** Mesh, Abdominoplasty, Pull-through, Retrorectus

## Abstract

**Purpose:**

Meshes clearly have improved outcomes for tissue approximation over suture repairs for incisional hernias. A knowledge gap exists as to the surgical complication rate and post-operative outcomes of a mesh rectus diastasis repair with a narrow well-fixed mesh that simultaneously narrows the rectus muscles and closes the widened linea alba.

**Methods:**

Inclusion criteria for mesh abdominoplasty were patients who (1) underwent a retrorectus planar mesh for repair of rectus diastasis (2) did not have a concurrent incisional hernia and (3) underwent skin tailoring as part of a cosmetic aspect of their care. The primary endpoint was surgical site occurrence (SSO) at any time after surgery as determined with review of their office and hospital medical records. Secondary endpoints included the length and complexity of the return to the operating room for any reason, non-surgical complications, readmission, post-operative recovery, surgical site infection, recurrence/persistence of abdominal wall laxity, and soft tissue revision rates.

**Results:**

SSO rate was 0% for the 56 patients who underwent this procedure. There were 40 women and 16 men. Superficial infections requiring oral antibiotics were required in three patients. One was a drain site erythema, one was for a superficial stitch abscess, and the third was for a mesh strip knot infection 6 months after the procedure. One patient underwent further tightening of the abdominal wall. Rates of soft tissue revision in the office for improved cosmesis were 23% in women and 6% in men.

**Conclusion:**

Repair of rectus diastasis with a narrow well-fixed mesh and concurrent skin abdominoplasty is a well-tolerated and reliable procedure with low recurrence and low SSO in the manner described. It is a procedure that works for both female and male pattern rectus diastasis, and has become our procedure of choice for moderate and severe rectus diastasis.

**Supplementary Information:**

The online version contains supplementary material available at 10.1007/s10029-021-02461-1.

## Introduction

Midline hernia repairs are surgical approximations of the medial borders of the rectus muscles. The same type of tissue approximation is required for the repair of rectus diastasis. The tissue type—namely the medial border of the rectus muscles—is the same for both conditions of incisional hernia and rectus diastasis. The deforming forces are the lateral three abdominal wall muscles and the centrifugal forces of the abdominal viscera and are the same for both conditions. The only difference between surgical corrections of midline incisional hernias and rectus diastasis is that in the latter condition, the posterior sheath does not need to be repaired.

In the classic study by Luijendijk [1] and in the follow-up manuscript by Berger [2], the repair of incisional hernias was found to be more complete and more reliable when meshes are used in comparison to sutures alone. The mechanism of failure leading to incisional hernia recurrence and loss of abdominal wall tightening is suture pull-through. Excessive tension on the sutures required for tissue approximation can lead to acute tearing of the tissues (dehiscence) or else the creation of scar that weakens over time from the forces applied and the development of an incisional hernia [3]. Scar is approximately 70% as strong as the underlying tissue type, and in many cases the scar is not strong enough to achieve a durable repair–hence the failure rates seen with incisional hernia repair with simple sutures [4]. Meshes distribute forces in the short term to limit suture pull-through. Over time, the filaments of meshes permit fibrovascular incorporation. The chronic foreign body response serves to create a scar scaffold that ensures that the tissue approximation will remain durable no matter the forces applied.

If incisional hernia repair and rectus diastasis repair are so similar, why is suture plication so common and meshes used so infrequently for linea alba stretching? Suture plication is commonly performed in patients with compliant abdomens, and so the deforming forces perhaps are not as strong. Suture plication is effective in most cases of diastasis, with low complication and recurrence rates [5]. However, there are many women with severe rectus diastasis from repeated pregnancies and twin gestations. There are patients with high demands on their torso for work or sports that request repair of rectus diastasis. There are patients with elevated body mass indices (BMI) where the suture tensions for repair are increased. Finally, what should be done for rectus diastasis and suboptimal cosmetics in men? Most of the studies on rectus diastasis suture plication and recurrence rates focus on women and not men, with a recent study enrolling 87 women but only 2 men [6]. Should the standard suture plication principles created for low demand women with mild to moderate rectus diastasis be applied to these cases? There is a reluctance of surgeons to use mesh, perhaps fearing the combination of open surgery with large pieces of foreign material. Furthermore, reviews on complex revision abdominoplasty do not even mention the word mesh [7]. Alternatives to a standard Pitanguy abdominoplasty [8] include minimally invasive techniques for linea alba plication and/or mesh placement for rectus diastasis. While appropriate for patients without skin excess, some have found that these minimally invasive techniques without additional skin resection can lead to unacceptable complication rates [9].

To fill the knowledge gap on the surgical outcomes of the use of mesh for aesthetic abdominoplasty, we report our experience. This is a follow-up and larger report of the senior author, who has published on the outcomes of mesh abdominoplasty with a 6% surgical site occurrence rate and no clinical recurrences in 32 male and female patients with both incisional hernias and rectus diastasis [10].

## Methods

A chart review of all patients undergoing aesthetic abdominal wall surgery using mesh between 2007 and 2018 performed by the senior author (G.D.) was conducted for this retrospective cohort study. Inclusion criteria were patients who had a narrow well-fixed retrorectus mesh to repair their abdominal wall defect, did not have an incisional hernia or require intra-abdominal dissection, and had aesthetic skin removal (that the patient paid for) as part of the procedure. The indications for surgery were for aesthetics, treatment of small ventral hernias, midline abdominal pain, and/or improved core function. The identified patients’ charts were analyzed for patients’ demographics, clinical characteristics, and for post-operative outcomes. Extracted clinical characteristics were history of smoking or diabetes, body mass index (BMI), and width of rectus diastasis. The primary endpoint was surgical site occurrence (SSO) at any time after surgery. We defined SSO by the Ventral Hernia Working Group definition of a deep wound infection, a wound dehiscence, a seroma, or the development of an enterocutaneous fistula within 30 days of the procedure [11]. Secondary endpoints included the return to the operating room for any reason, non-surgical complications, readmission, post-operative recovery as assessed by the number of clinic visits within 6 months, superficial surgical site infection not requiring an incision and drainage, recurrence/persistence of abdominal wall laxity, and soft tissue revision rates. This study was approved by the Northwestern University Institutional Review Board.

## Patient evaluation and decision-making

After a thorough general history and physical examination as well as a focused abdominal wall examination, a decision is taken as to the appropriateness of a mesh repair of rectus diastasis. All candidates must be able to undergo a 2–3 h procedure, expect a post-operative hospitalization for pain, and are willing to undergo a 3–6 week recovery phase. All patients are told that they can have severe complications, including chronic pain, deep vein thrombosis, pulmonary emboli, and death. Exclusion criteria include BMI greater than 35, poor cardiac or pulmonary status that would prevent walking up several flights of stairs or the inability to lie flat, and bleeding disorders. Smoking was a relative contraindication. Decision-making is analog and not digital, meaning that there are numerous factors that lead both the patient to desire surgery and the surgeon to offer a mesh repair as opposed to a suture plication of the linea alba (Table 1). Patients who undergo mesh repairs are good surgical candidates with moderate to severe rectus diastasis with a generalized loss of abdominal wall tone between their semilunar lines, and have high demand on their torso for physical activity. As the loss of abdominal tone between the semilunar lines is a summation of both rectus diastasis and rectus muscle widening, there is no minimum width of rectus diastasis for patients to be treated with this technique. However, most patients have rectus diastasis greater than 4 cm in transverse dimension.

**Table 1:** Decision-making for mesh repair of rectus diastasis versus suture plication

Suture plication	Well-fixed narrow mesh
Mild rectus diastasis	Severe rectus diastasis
2–3 cm of rectus diastasis	5 cm or greater rectus diastasis
Localized abdominal wall stretching at linea alba	Generalized abdominal wall stretching between semilunar lines
Rectus muscles each about 6 cm wide	Rectus muscles wider than 7–8 cm wide
Good tone	Floppy abdomen
Female	Male
No scars	Already has a vertical scar
No weight loss	Massive weight loss, or with elevated BMI (men)
Low demand physical activity	High demand
Fear of mesh	Acceptance of mesh
Initial procedure	Revision abdominoplasty

## Surgical technique

### Skin abdominoplasty

#### Standard incision in the majority of females

Skin handling for the low transverse incision is done in the style of Pitanguy. An incision is made 6–7 cm cephalad from the introitus, and the skin is elevated widely with cautery (Peak Plasmablade, Medtronic, Minneapolis MN) up to the umbilicus. The umbilical stalk is delivered from the abdominal flap, and the epigastric skin is dissected off of the stretched out abdominal wall predominantly with blunt dissection to leave the linea alba intact. Dissection first laterally and then moving to the midline helps the surgeon to remain in the proper plane. Above the umbilicus, the skin is elevated at least to the semilunar lines for placement of the mesh and for skin redraping. Conservative liposuction in the high epigastric area is performed when indicated. The low midline skin up to the umbilicus is incised for exposure. After placement of the mesh, the bed is placed into 45 degrees reflux, and the umbilical stalk skin is brought out through the abdominal skin flap. Two drains are placed, and the skin is closed with 3–0 polyglactin braided suture for the deep dermis and 4–0 monofilament polyglactin suture for the superficial dermis. The drains are removed when the drainage is less than 30 cc from each. Drains typically come out within one week. Other than a perioperative dose, no additional antibiotics are given. The tissues are irrigated with a dilute antibiotic solution during the procedure.

#### Vertical incision in some females and all males

While the best scar is one that can be easily hidden, it is also true that many patients with suboptimal cosmesis of the abdomen from rectus diastasis always wear shirts and would strongly consider a vertical incision if it would provide a better overall abdominal contour. In all surgeries with an aesthetic component, scars should be placed when possible between aesthetic subunits [12], and the subunit of the abdomen is in the vertical midline. Like the decision whether to use mesh or to perform a suture plication for abdominal wall tightening, the placement of the incision is a complex decision that encompasses many factors as detailed in Table 2. A minority of patients (six women and all of the men) had vertical skin incisions for their mesh abdominoplasty. These six patients either had pre-existing midline scars, prior abdominoplasty rendering a repeat procedure more difficult, surgery that required the removal of previously placed uncomfortable umbilical hernia mesh, or else had severely stretched epigastric skin that would be difficult to remove via a low transverse incision. All of the men had a vertical incision, as the scar is hidden by hair-bearing skin and the physical examination finding that men do not typically have excess skin in the hypogastric area. Additionally, vertical incisions allow for creation of an umbilicus using "pumpkin-teeth" flaps,[13] and the neo-umbilicus breaks up the vertical scar into an epigastric scar and a hypogastric scar. A short suprapubic transverse incision is typically required to prevent a dog-ear. The author prefers the vertical incision for the most severe cases of rectus diastasis over 8 cm wide and for men, as it provides for the best shape of both the abdominal wall and for the overall improvement of skin contours.

**Table 2:** Decision-making for standard low transverse versus vertical incision

Low transverse incision	Vertical incision
Women	Men
Hypogastric skin excess	Epigastric skin excess
No scars	Pre-existing vertical scar
No umbilical hernia	Umbilical hernia
Moderate rectus diastasis	Severe rectus diastasis
More skin elevation	Less skin elevation
Less risk adverse	More risk adverse
Short torso	Long torso
	Prefers to walk straighter after surgery
	Always wears a shirt
	Massive weight loss
	Revision abdominoplasty
	Need to remove prior umbilical hernia mesh

### Rectus diastasis repair with a narrow well-fixed mesh

**Fig. 1:**
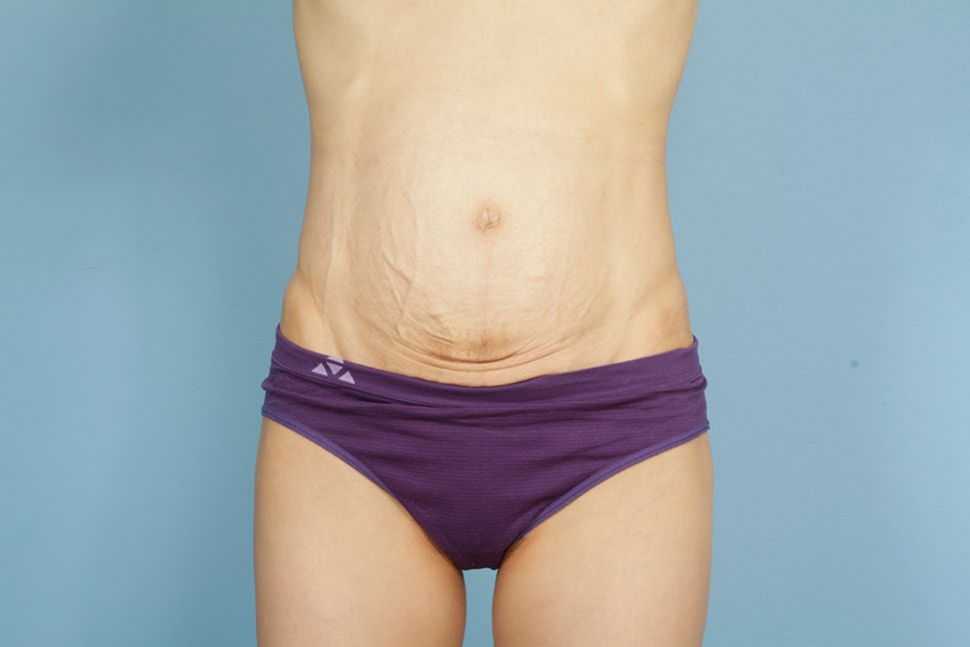
Preoperative photo of slender woman with 6 cm rectus diastasis and widened rectus muscles

**Fig. 2:**
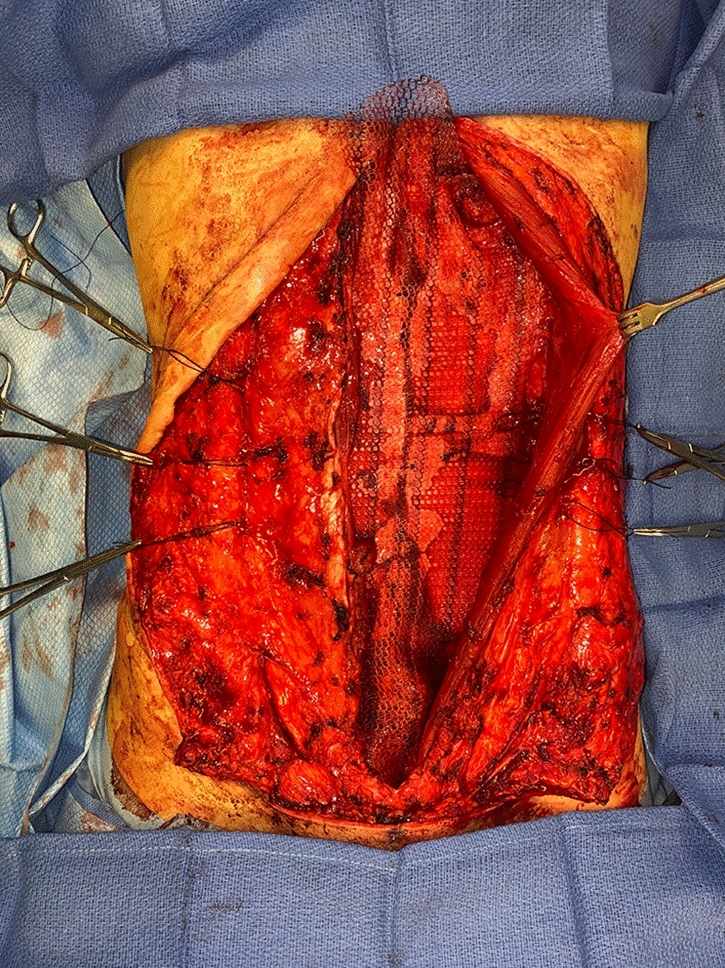
Intraoperative photo of narrow polypropylene mesh inset with transfascial sutures

**Fig. 3:**
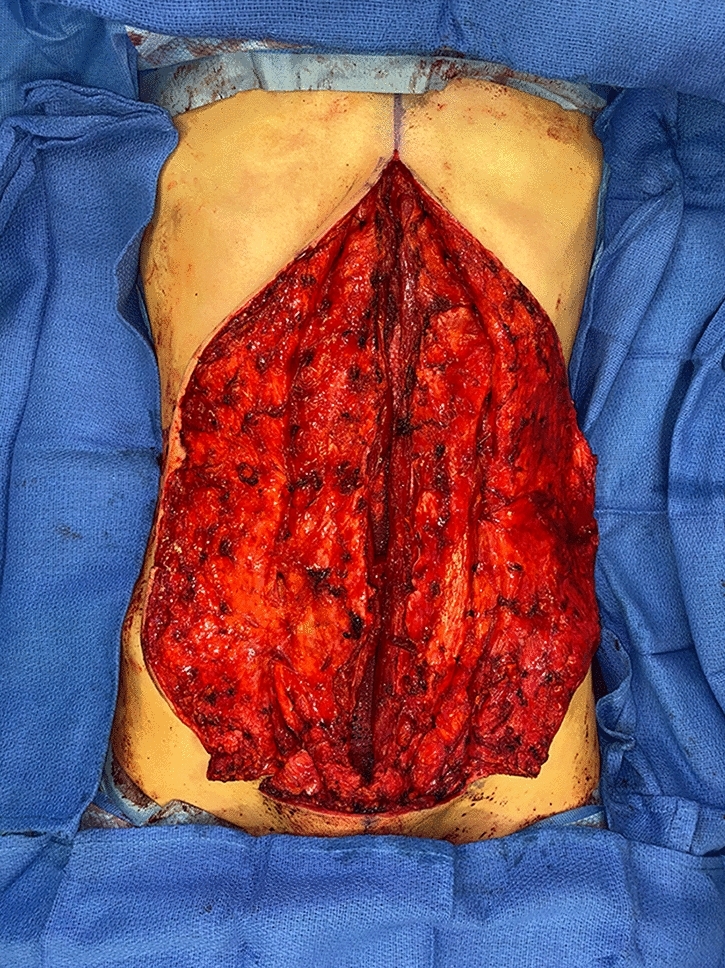
Mesh completely inset, just before closure of the medial border of the rectus muscles. The laterally placed sutures serve to narrow the rectus muscles and emphasize the semilunar lines

**Fig. 4:**
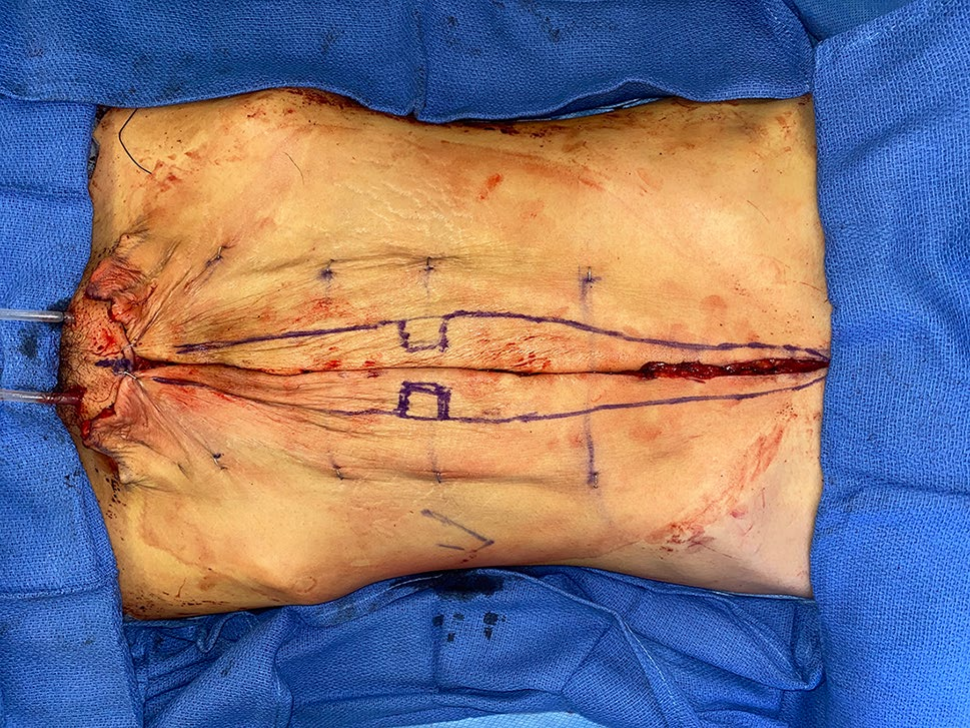
Skin is excised both medially and inferiorly. Staples are placed prior to the initial incision as landmarks so that skin is excised equally from both hemiabdomens. “Pumpkin-teeth” flaps are drawn. A drawn line on the skin is the xyphoid

**Fig. 5:**
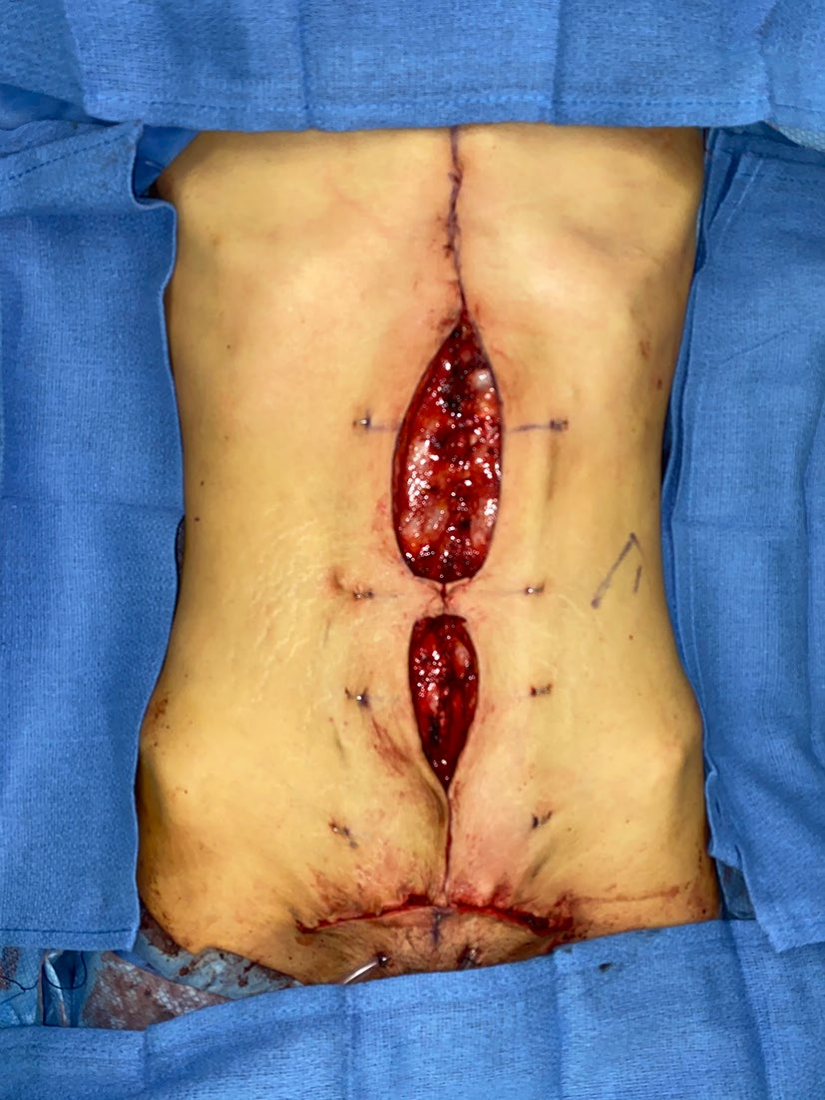
“Pumpkin-teeth” flaps are tacked down to the abdominal wall as first step in creating a neo-umbilicus

**Fig. 6:**
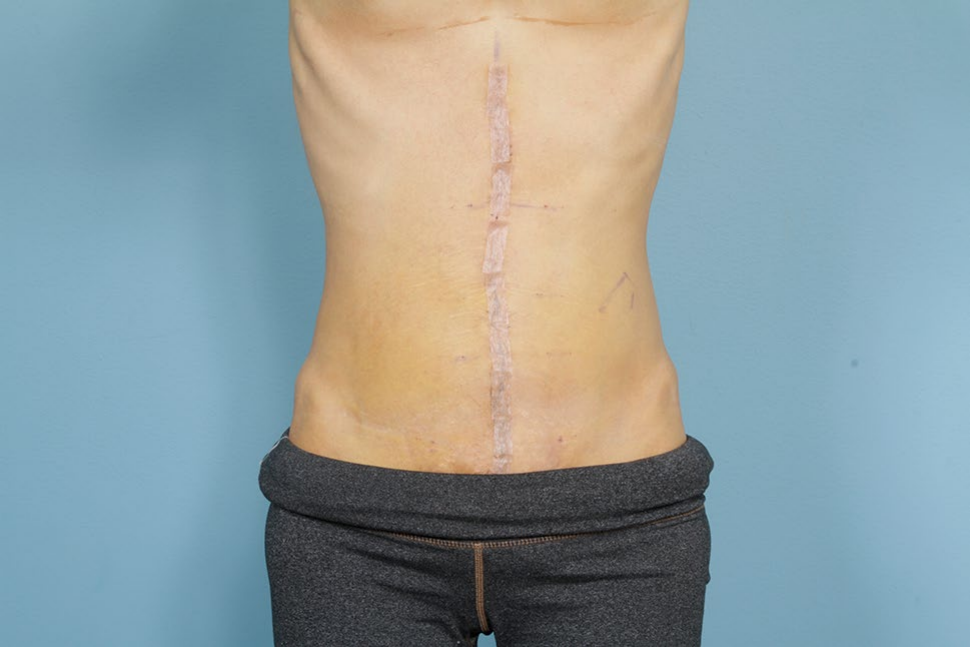
Early post-operative appearance

**Fig. 7:**
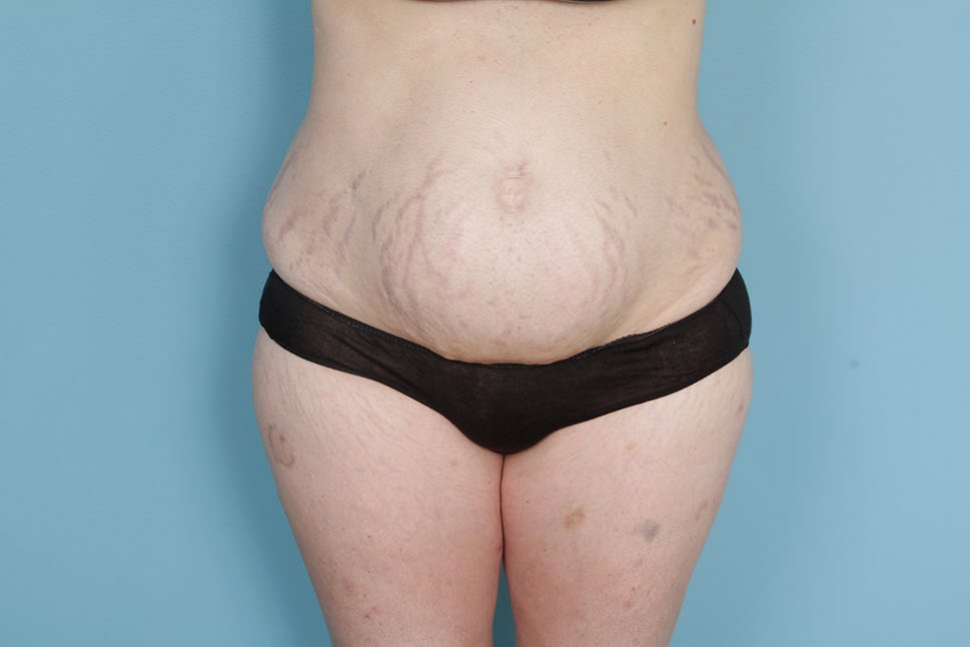
Preoperative view of female patient undergoing mesh abdominoplasty through a transverse incision

**Fig. 8:**
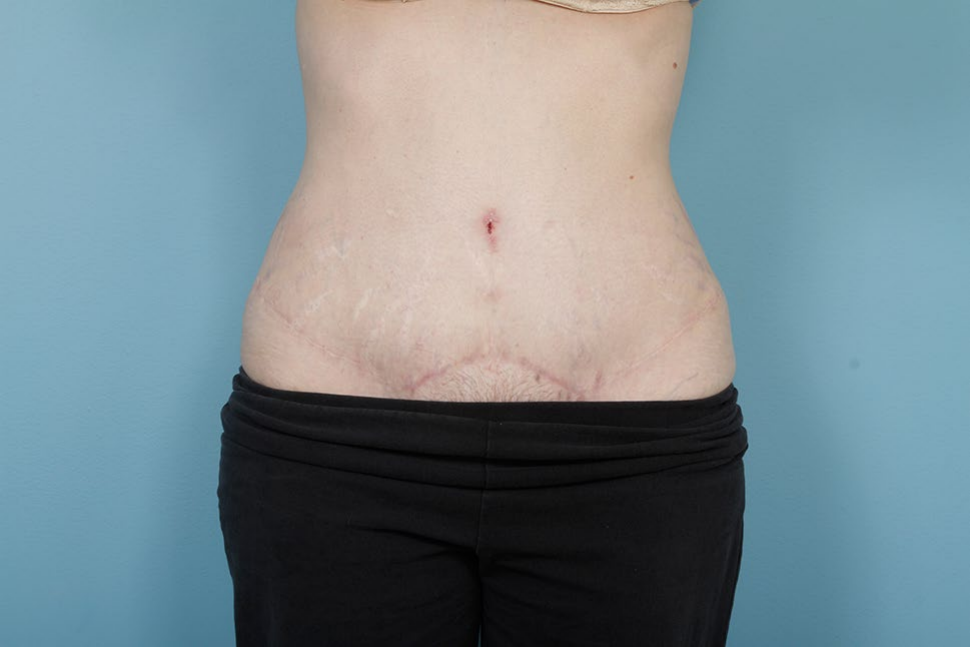
Post-operative 1 year view of female patient who underwent a mesh abdominoplasty through a transverse incision

No matter the skin incision, the anterior rectus sheath is opened on its medial border to expose the rectus muscle. The muscle is bluntly freed from the underlying posterior rectus fascia from the xyphoid to several centimeters below the umbilicus where these two fascial incisions are joined and the Space of Retsius is entered. It is important to remain extra-peritoneal for this dissection. Typically, a small blood vessel from the deep inferior epigastric artery (DIEA) is identified traveling to the umbilicus, and this is preserved. An uncoated mid-to lightweight macroporous polypropylene mesh, (Soft Prolene, Ethicon, New Brunswick NJ) is cut to fit into the space, with its widest dimension being 10–11 cm transversely in the supraumbilical area. The key to this procedure is the placement of transfascial sutures that pass through the anterior rectus fascia and muscle near the semilunar lines, grab a small element of the mesh as a "U" bite, and then return back through the muscle and anterior rectus fascia. 0-polypropylene sutures are typically used for securing the mesh, but the thinnest patients receive 0-polydiaxanone sutures to limit palpability. The sutures are placed long and snapped for later tying. Full visualization of the undersurface of the rectus muscle is necessary to avoid encircling an intercostal nerve that could cause long-term pain. A tongue of mesh is placed into the Space of Retsius without fixation, both for the functional reason that tension needs to be greatest in the epigastrium, as well for the practical reason to avoid a puncture of the DIEA. Eight or nine sutures a side are placed for each hemi-abdomen. These multiple sutures place the tension on the mesh to close down the linea alba, and to narrow the rectus muscles to a pre-pregnancy "ideal" of 6 cm wide [14]. Half of the mesh (about 5.5 cm) underlies the left rectus, and half (about 5.5 cm) underlies the right rectus muscle–angling on the trajectory of the suture makes up for the final half centimeter on each side. Above the immediate supraumbilical area and with the narrowing of the midline from the rib cage, the mesh is cut to fit and narrowed. The sutures are placed as cephalad as possible. Immediately before closing, an assessment of umbilical stalk viability is made. If one or both of the small feeder vessels to the posterior sheath is in continuity, then a hole is made in the mesh for the umbilical stalk to emerge. With the patient fully muscle relaxed, the abdominal wall is then closed with tying down of the snapped lateral sutures. Though the closure may seem tight and under tension, this style of abdominal wall closure is tolerated by the tissues and has been reliable without any recurrences for either rectus diastasis or incisional hernia repair with a documented 2-year follow-up [15]. In the epigastrium for women, the medial borders of the rectus are tacked down to the mesh to create two separate rectus muscles, while this is not performed in the hypogastrium. Strips of mesh are used for the midline closure in patients with thick skin flaps [16]. Other than occasionally in the area of the xyphoid, all of the mesh is covered by the rectus muscles. Figure 1 and Video 1 demonstrate the preoperative rectus diastasis in a slender woman. Figures 2, 3, 4, 5 and 6 illustrate the technical steps of a mesh abdominoplasty through a vertical incision (it is easier to photograph the technical details when the skin is fully elevated in this manner). Figures 7 and 8 illustrate the long-term results possible.

## Results

Results are presented in Table 3. Of note is the low rate of smoking in these patients, the average width of the rectus diastasis of 6–7 cm, a 2.5 day hospitalization, and 2–3 clinic visits in the first 6 months after surgery. There was a 0% SSO rate, though three patients had superficial infections that resolved with either oral antibiotics or the removal of a mesh strip knot as an office procedure 6 months after the procedure at the site of a neo-umbilicus in a man. There were no rehospitalizations, and ten patients had soft tissue revisions as office procedures for improved aesthetics. One patient complained of localized abdominal wall pain at 6 months after surgery that resolved with physical therapy. One patient underwent retightening of her abdominal wall after a mesh abdominoplasty. The tentative diagnosis for this patient was new stretching of the abdominal wall lateral to the semilunar lines. A recent report documented the aesthetics of this procedure for female patients [17]. Patients with severe rectus diastasis have worse preoperative aesthetic scores than do patients with milder diastasis who are undergoing suture plication. Both groups have similar improvements in aesthetic scores, but the suture plication group starts with improved aesthetics and remains aesthetically more pleasing than the mesh group as judged by four blinded observers.

**Table 3:** Data from 56 patients undergoing retrorectus mesh repair with a well-fixed narrow mesh

Demographic characteristics		
Sex	Male	Female
Total no. of mesh abdominoplasty patients	16	40
Age (mean, range)	57 (35–74)	42 (29–70)

Clinical characteristics	Mesh abdominoplasty (*n* = 16)	Mesh abdominoplasty (*n* = 40)
BMI (kg/m^2^) (mean, range, standard deviation)	29 (24–37) (4.0)	26 (18–40) (5.3)
Smoking status		
Current (*n*, %)	2 (12%)	0 (0%)
Former (*n*, %)	1 (6%)	0 (1%)
Never (*n*, %)	13 (81%)	40 (95%)
Diabetes		
Yes (*n*, %)	3 (19%)	0 (4%)
No (*n*, %)	13 (81%)	40 (96%)
Concomitant hernia (patients) (*n*)		
Epigastric	9	9
Umbilical	7	13
Width of rectus diastasis (cm) (mean, range)	6 (2–8)	7 (4–15)
Follow-up visits in first 6 months (mean, range)	2 (1–6)	3 (1–7)
Hospital stay (days) (mean, range)	2.5 (0–5)	2.5 (0–6)
SSI (*n*, %)	2 (12%)	1 (3%)
SSO (*n*, %)	0 (0%)	0 (0%)
Other complication	Drain site erythemia, infected mesh strip knot at umbilicus 6 months after procedure	Superficial suture abscess, resolved with dermal knot removal and antibiotics
Hospital readmission (*n*, %)	0 (0%)	0 (0%)
Muscle revision (*n*, %)	0 (0%)	1 (3%)
Office soft tissue revision (*n*, %)	1 (6%)	9 (23%)
Follow-up	73 weeks	45 weeks

## Discussion

Surgical repair of rectus diastasis requires that the circumference of the abdominal wall be decreased in the axial plane. This requires the forceful placement of tension by the sutures on the tissues across the mesh to narrow the distance between the semilunar lines. While a suture plication of the linea alba is successful for mild or even moderate diastasis for patients with compliant post-partum abdominal walls, this may not be true for severe rectus diastasis or for patients (such as men) with high tensile demands on their tissues. The greater the tension required to achieve a meaningful correction, the greater the tendency for a standard suture-only plication to pull-through, resulting in a surgical failure over time. The anterior rectus fascia (which is necessary for suture plication techniques) is itself stretched and possibly prone to suture pull-through. Level one data for hernia repair exists that sutures under tension fail more often than do meshes that can distribute forces and provide a scar scaffold for healing. Our procedure employs transfascial sutures and a narrow mesh to anatomically decrease the circumference of the abdominal wall and simultaneously narrow the rectus muscles, but without folding or plicating the rectus muscles inwards that is associated with all suture plication techniques. These sutures are under high tension, and it requires forceful pulling to tighten the musculature. This tension is tolerated, however, because it is distributed amongst many points of fixation.

This mesh abdominoplasty procedure is performed using long incisions for visualization as well as for the ability to resect and tighten skin. It stands to reason that the greater the circumference decrease in the abdominal wall, the more that skin will become redundant. These patients all have paid money out of pocket to achieve a more youthful abdominal wall, and muscle tightening alone with a residual loose skin envelope often does not suffice. Scars are left within the underwear line in women, and occasionally in the most severe cases in the aesthetic subunit of the vertical midline. The latter approach tightens the flanks for improved contours. The 0% SSO in this series is lower than the SSO senior author's series in suture plication standard abdominoplasties [17]. One explanation for this observation is that the soft tissues have improved healing when on top of a stable abdominal wall platform. The well-fixed nature of the mesh may be significantly protective against deep hematomas and fluid collections. The deep position of the mesh, maintenance of tissue blood flow, use of the PlasmaBlade, drains, antibiotic irrigants, and blunt dissection all contribute to this low SSO.

The procedure described in this manuscript is quite different from the use of a minimally fixed retromuscular mesh that does not emphasize tension placed across the mesh in the axial (or transverse) plane. Large unfixed polypropylene meshes up to 24 cm wide [18] do indeed provide tissue support, but any decrease in circumference of the abdominal wall requires that all of the tightening occur at the medial border of the rectus muscles where excessive tension may lead to suture pull-through. Large unfixed meshes do not decrease the width of a post-partum rectus muscle (the dissection and tension at the midline could theoretically widen the muscle), nor do they create an emphasis of the semilunar line. Alternatives to mesh abdominoplasty include suture plications that may result in suture pull-through, minimally invasive techniques that do not generate high tension in the axial plane across the mesh and do not involve the excision of skin, and overlay meshes that are fixated with multiple sutures but are prone to seroma formation. Placement of transfascial sutures is not a widely performed technique, perhaps for the presumed difficulty involved in placing the sutures while avoiding intercostal nerves and the deep inferior epigastric artery. While short-term pain typically requires a hospitalization, long-term pain has not been an issue for these patients, though one patient required physical therapy at 6 months. If chronic pain were to develop, treatment would involve intercostal nerve excision and allograft reconstruction [19]. This has not been necessary to date.

In our hands, a mesh abdominoplasty using a well-fixed retromuscular mesh is associated with low SSO and a durable improvement of abdominal shape and contour in both men and women. Technical details in placement of the mesh construct may be an important factor in achievement of these outcomes.

## Supplementary Information

Below is the link to the electronic supplementary material.

Supplementary file1 Video of patient of figure 1 doing a sit-up maneuver to demonstrate rectus diastasis (MOV 9475 KB)

## Declarations

### Availability of data

Full, de-identified, upon request.
